# Endoplasmic Reticulum-Dependent Apoptotic Response to Cellular Stress in Patients with Rheumatoid Arthritis

**DOI:** 10.3390/ijms26062489

**Published:** 2025-03-11

**Authors:** Aleksandra Kucharska-Lusina, Maciej Skrzypek, Agnieszka Tokarczyk, Grzegorz Dragan, Ireneusz Majsterek

**Affiliations:** Department of Clinical Chemistry and Biochemistry, Medical University of Lodz, 92-215 Lodz, Poland

**Keywords:** endoplasmic reticulum stress, unfolded protein response, PERK, apoptosis, rheumatoid arthritis

## Abstract

Rheumatoid arthritis (RA) is a chronic, common autoimmune disease. It is characterized by inflammatory polyarthritis, which can lead to permanent disability in patients. Current treatment is mainly symptom-related, aiming to reduce pain and inflammation, but does not lead to a full recovery. This treatment includes non-steroidal anti-inflammatory drugs (NSAIDs) and disease-modifying anti-rheumatic drugs (DMARDs). It has been shown that, due to chronic inflammation, reduced glucose levels and hypoxia, endoplasmic reticulum (ER) stress is induced in RA patients, leading to the activation of multiple signaling pathways, including the ER-dependent adaptation of the unfolded protein response (UPR) pathway. The aim of this study was to assess the level of apoptosis in patients diagnosed with RA. The study sought to investigate whether UPR response correlated with apoptosis induction could serve as a potential diagnostic marker or therapeutic target. In vitro studies have shown that UPR pathway activity can be observed in patients diagnosed with RA. The study group consisted of PBMC cells from 61 individuals, including a total of 31 rheumatoid arthritis patients and 30 healthy controls. In order to validate UPR activation, we estimated molecular markers of ER stress via RT-qPCR expression analysis. *GAPDH* expression was used as a standard control. Elevated levels of mRNA for the *eIF2α* (*p*-value = 0.001903), the *BBC3* (*PUMA*) (*p*-value = 0.007457 × 10^−7^) and the *TP53* (*p*-value = 0.002212) were confirmed in a group of RA patients. Further analysis showed that after the induction of apoptosis the percentage of DNA contained in the tail was 37.78% higher in RA patients than in the control group (*p*-value = 0.0003) measured by comet assay. The exogenous damage caused by hydrogen peroxide was found to be statistically elevated in RA patients and the caspase-3 level was calculated of 40.17% higher than in controls (*p*-value = 0.0028). It was also found that PBMC cells from RA patients were more sensitive to apoptotic induction. Our results were confirmed by flow cytometry. The most important finding from our data was the confirmation of elevated sensitivity to apoptosis induction in RA patients; the results showed a 40.23% higher percentage of cells in early apoptosis than in the control group (*p*-value = 0.0105). Our results may help to assess the feasibility of the application of early diagnosis and targeted therapy in the treatment of RA patients, including the ER signaling pathway via selected UPR-dependent molecular inhibitors.

## 1. Introduction

Rheumatoid arthritis [RA] is one of the most common autoimmune diseases. It is estimated to occur in approximately 1% of the population, with women being much more commonly diagnosed with this disease. The main symptoms include bone erosion, synovitis of the joints and pannus formation. Progressive swelling and joint tenderness can result in permanent disability for patients. RA is characterized by the presence of autoantibodies, including anti-citrullinated protein antibodies (ACPAs) and rheumatoid factor (RF) [1,2,3]. The interaction of differentiated immune cells with fibroblasts and cytokines leads to the development of inflammation in the synovial tissue. In addition, chronic systemic inflammation can lead to damage to lung tissue, the nervous system, the vascular system, heart tissue, or the kidneys [3].

The pathogenesis of RA is based on the interaction of genetic and epigenetic factors, as well as environmental, immunological, metabolic and microbiological factors. It has been shown that disease progression is determined by interactions between immune cells, including T lymphocytes, B lymphocytes, dendritic cells, neutrophils, monocytes, macrophages, mast cells and fibroblast-like synoviocytes. Dysfunction of immune cells and signal transduction pathways adversely affects tissue repair, resulting in organ damage, mainly to joints [4]. It is important to emphasize that the mechanisms by which immune cells mediate the development of synovitis and cartilage erosion are not present in all patients, and the severity of these symptoms is dependent on the stage of the disease [3]. Genetic studies show that an increased risk of developing RA is associated with more than 100 genetic polymorphisms. In addition, environmental factors may activate innate immunity, resulting in the modification of protein antigens, thereby impairing self-tolerance. It should be emphasized that the development of RA is a long-term process that can last almost the entire life of the patient. It begins with a phase of asymptomatic autoimmunity involving the interaction of autoantibodies with post-translationally modified proteins, such as citrullinated antigens, which can last for many years. This is followed by symptomatic acute synovitis, which can develop into chronic synovitis resulting in joint destruction. At the same time, pannus formation occurs, described as an abnormal wound healing process, which can result in irreversible damage to bone, cartilage and tendons, as well as tissues [4]. 

The accumulation of misfolded proteins in the endoplasmic reticulum (ER) activates the unfolded protein response pathway (UPR), which activates ER-associated degradation (ERAD). ERAD is a mechanism responsible for the identification and transport of misfolded ER proteins to the cytosol for proteasomal degradation. The mechanism explaining substrate recognition by ERAD in mammals still leaves one with many questions. The action of the UPR is based on the three major ER transmembrane receptors: protein kinase-like ER kinase (PERK), protein inositol requiring 1 (IRE1α) and activating transcription factor 6 (ATF6) [5,6]. These proteins are characterized by the presence of ER luminal domains, which have the ability to detect unfolded proteins. They also contain cytosolic regions that protect the cell from ER stress under physiological conditions via the transcriptional, translational apparatus or by interacting with signaling molecules as a scaffold [7]. UPR-regulated proteins are also present in the ER that possess the unfolded protein response element (UPRE) or ER stress response elements (ERSEs), which are sequences that activate transcription due to increasing amounts of unfolded proteins. When the amount of unfolded proteins is too much of a burden for the cell and cellular adaptation as a result of the UPR is insufficient, an apoptotic response initiated by the protein c-Jun amino-terminal kinase (JNK) is activated [5,8].

As has been mentioned, when the amount of misfolded proteins is too great of a burden on the UPR, apoptosis is activated in the cell. There are many potential mechanisms to explain the process of initiation and control of ER stress-induced apoptosis. The activation of the apoptotic pathway results in the activation of Bcl-2 family proteins in the mitochondria [5]. These proteins are referred to as the main regulators of apoptosis. These include pro-apoptotic proteins such as BAX and anti-apoptotic proteins such as Bcl-2 [6]. Studies show that increased expression of the EIF2AK3 gene, which codes the PERK protein and phosphorylated eukaryotic translation initiation factor 2 subunit-α (eIF2α), is observed in the macrophages and synovial tissues of patients diagnosed with RA. Moreover, the initiation of inflammation in patients with RA may be influenced by the PERK/eIF2α UPR transduction pathway, resulting in the activation of the apoptotic pathway [9].

The aim of the study was to assess the level of apoptosis in patients diagnosed with RA. The study sought to investigate whether specific molecular markers, including selected genes specific for UPR response correlated with apoptosis induction, could serve as potential diagnostic markers or therapeutic targets. The results may help to assess the feasibility of applying early diagnosis and targeted therapy in the treatment of patients with RA.

## 2. Results

### 2.1. Measurement of Percentage of Living Cells 

The measurement of the percentage of live cells in isolated PBMC samples did not show statistically significant differences between RA patients and controls (*p*-value = 0.3146). The average percentage of living cells in RA patients was 93%, and that in the control group was 94%.

### 2.2. Gene Expression

**Figure 1:**
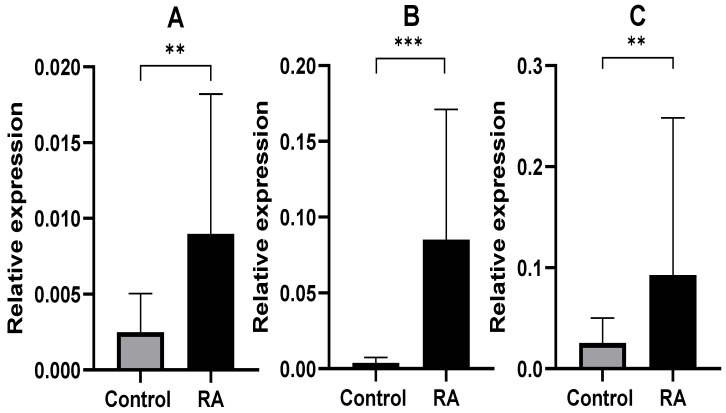
Relative expression genes in the blood of rheumatoid arthritis patients and controls: (**A**) eIF2α gene expression in patients and healthy groups; (**B**) BBC3 gene expression in patients and healthy groups; (**C**) TP53 gene expression in RA patients and healthy groups. Data are presented as means ± SEM (** *p* < 0.01 and *** *p* < 0.0001).

Gene expression analysis was performed for four genes, including the endogenous control gene GAPDH and three test genes, namely eIF2α, TP53 and BBC3. Considering the median, it was found that the expression of eIF2α, TP53 and BBC3 (PUMA) in the blood of RA patients was significantly increased compared with the control group. The results are shown in Figure 1. The *p*-value for the eIF2α gene was 0.001903, the *p*-value for the BBC3 gene was 0.007457 × 10^−7^, and the *p*-value for the TP53 gene was 0.002212.

### 2.3. Comet Assay

**Figure 2:**
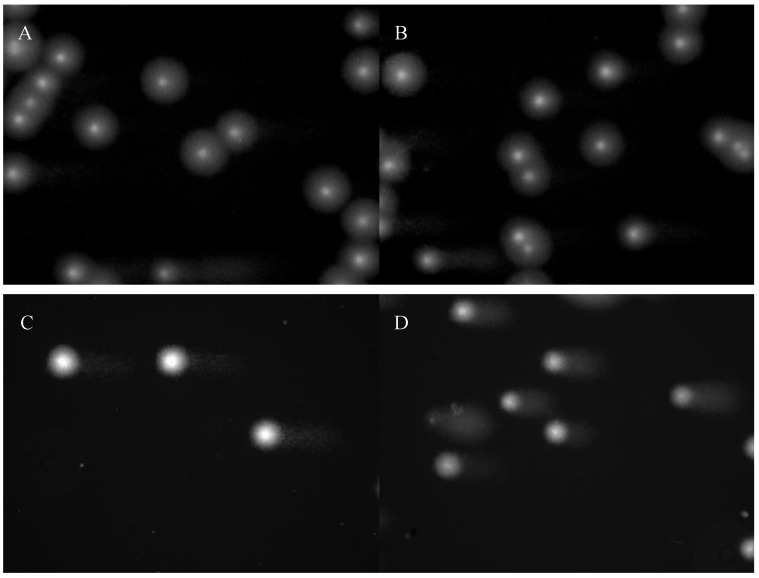
The microscopic images for the comet assay: (**A**) endogenous damage in controls; (**B**) endogenous damage in patients with RA; (**C**) induction of apoptosis by hydrogen peroxide in controls; (**D**) induction of apoptosis by hydrogen peroxide in RA patients.

**Figure 3:**
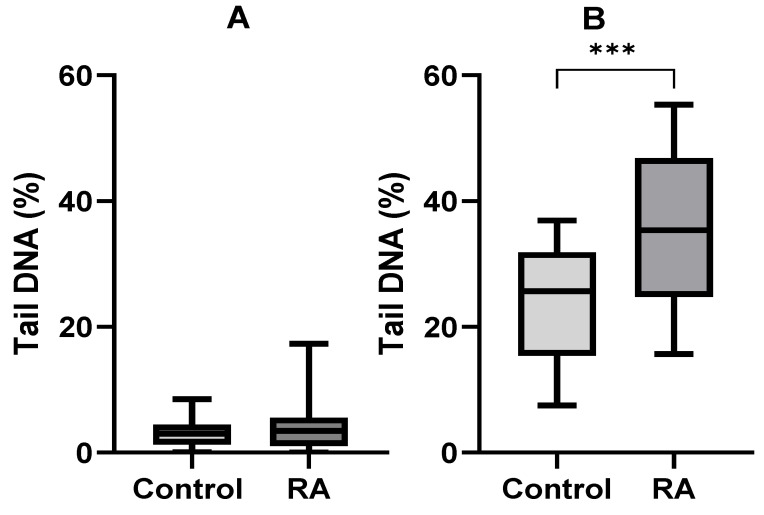
Percentage of DNA contained in the tail of RA patients and controls: (**A**) percentage of the tail DNA in endogenous damage; (**B**) percentage of the tail DNA after the induction of apoptosis by hydrogen peroxide. Data are presented as means ± SEM (*** *p* < 0.001).

The microscopic images during the comet assay analysis are presented in Figure 2. The data of the comet assay did not indicate statistically significant differences in the percentage of tail DNA in the case of endogenous damage (*p*-value = 0.11). However, in RA patients the percentage of DNA in the tail after hydrogen peroxide incubation was 37.78% higher than in the control group (*p*-value = 0.0003), as shown in Figure 3.

### 2.4. Measurement of Caspase 3 Activity

**Figure 4:**
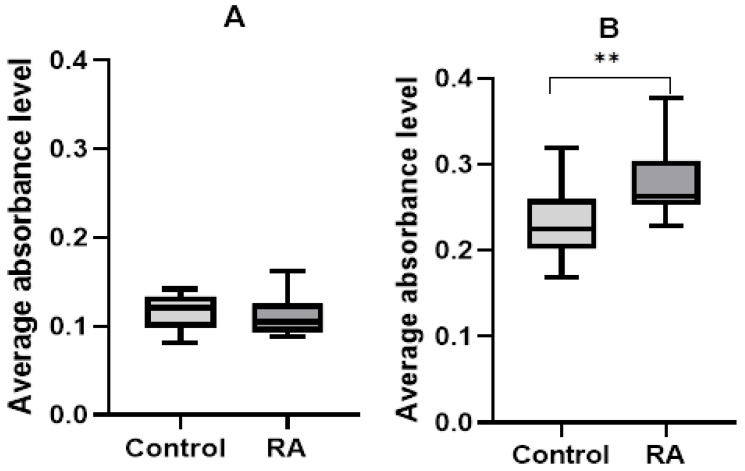
Relative activity of caspase-3 in the PBMC of rheumatoid arthritis patients and controls: (**A**) levels of caspase-3 caused by endogenous damage in patients and healthy groups; (**B**) levels of caspase-3 caused by exogenous damage induced by hydrogen peroxide in patients and healthy groups. Data are presented as means ± SEM (** *p* < 0.01).

The test for caspase-3 activity in cell suspensions did not show statistically significant differences in the case of testing for endogenous damage (*p*-value = 0.3727). However, the statistically significant differences in exogenous damage caused by hydrogen peroxide were estimated between patients and controls, reflecting the higher activity of caspase-3 for RA (*p*-value = 0.0028). Based on the results, it can be calculated that caspase-3 levels were 40.17% higher in RA patients than in controls in the case of exogenous damage, as shown in Figure 4.

### 2.5. Measurement of Apoptosis Using Flow Cytometry

**Figure 5:**
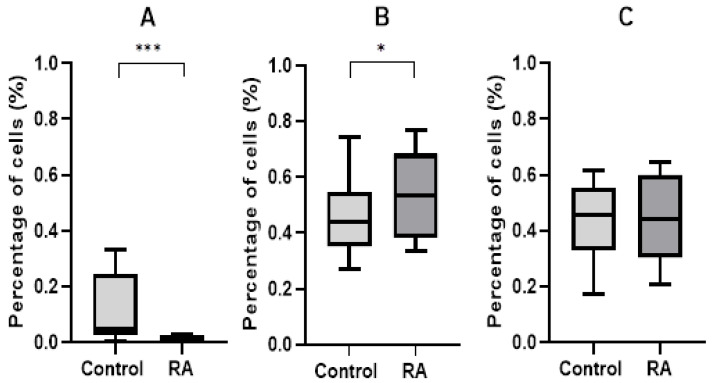
The relative percentage of cells in the population of peripheral blood mononuclear cells in RA patients and controls after the induction of apoptosis by hydrogen peroxide: (**A**) percentage of healthy cells in patients and healthy groups; (**B**) percentage of cells in early stage of apoptosis in patients and healthy groups; (**C**) percentage of dead cells in patients and healthy groups. Data are presented as means ± SEM (* *p* < 0.05 and *** *p* < 0.001).

In the case of cytometric measurements, statistically significant differences were demonstrated in the percentage of cells in the stage of early apoptosis and healthy cells between the group of RA patients and controls after the evaluation of exogenous damage caused by hydrogen peroxide. At baseline, our study showed no statistical differences between populations while examining endogenous damage (live cells—*p*-value = 0.7394; early apoptotic cells—*p*-value = 0.6775; and dead cells—*p*-value = 0.2121). However, the exogenous damage evaluation with hydrogen peroxide showed that the percentage of healthy cells was higher in controls than in RA patients (*p*-value = 0.0003) and the percentage of cells in early apoptosis was higher in the RA population than in the control population (*p*-value = 0.0105). In the case of the percentage of dead cells, no statistically significant differences were observed between both populations (*p*-value = 0.5391). Finally, an increase in cells of 40.23% was measured during the stage of early apoptosis in RA patients compared to healthy controls, as shown in Figure 5.

## 3. Discussion

The heterogeneity of the clinical manifestations of RA patients often results in a delayed visit to a rheumatologist and, consequently, in a later diagnosis and reduced effectiveness of treatment. In order to improve the diagnosis process, the European League Against Rheumatism (EULAR) and the American College of Rheumatology (ACR) released modified criteria for classifying symptoms in 2010 [10,11]. These can be divided into four groups: duration of symptoms, presence of an acute phase response (tests performed: C-reactive protein (CRP) or erythrocyte sedimentation rate (ESR)), joint assessment and serological test result (ACPA or RF). A score is assigned to each of these, and a final score of at least 6 out of 10 indicates the presence of definite RA. A score of less than six prevents a diagnosis of RA but such a patient should be monitored as there is a risk of worsening symptoms. It is important to note that a patient does not have to complete all tests to be diagnosed with RA. For example, irrespective of the serological test result or the acute phase response, with a sufficient number of affected joints and a duration of symptoms of at least 6 weeks, a patient may score six points, which is sufficient to make a diagnosis [12].

The treatment of RA has improved significantly since the invention of biological disease-modifying anti-rheumatic drugs (bDMARDs) [13]. At the end of the 20th century, the US Food and Drug Administration approved five tumor necrosis factor α (TNFα) inhibitors, including infliximab and etanercept. A CD80/86 inhibitor and an interleukin-6 (IL-6) receptor antibody inhibitor are also commercially available [14]. Between 2013 and 2020, a further five drugs were approved, including tofacinib, baricitinib, peficitinib and upadacitinib, and, most importantly, Janus kinase inhibitor. JAK inhibitors are intracellular or orally administered compounds of low molecular weight, and they are the only ones categorized as targeted synthetic DMARDs. Furthermore, bDMARDs have the ability to bind and inhibit the activity of extracellular proteins. In contrast, a JAK inhibitor has an inhibitory effect on intracellular JAK kinase activity. JAK is involved in the signaling pathway of class I/II cytokines, such as IL-2, IL-6, granulocyte macrophage colony-stimulating factor (GM-CSF), erythropoietin and interferon α (IFNα), which participate in immune and inflammatory responses. A JAK inhibitor binds to the JAK adenosine triphosphate binding site in a competitive manner, resulting in the inhibition of JAK enzymatic activity and therefore also inhibiting the activity of the mentioned cytokines. This ability distinguishes them from bDMARDs, which, as monoclonal antibodies, can inhibit the activity of only one specific cytokine. Studies have proven the role of certain type I/II cytokines in the pathophysiology of RA. In particular, the administration of anti-IL-6 receptor antibodies or anti-GM-CSF receptor α antibodies results in patients’ health improvement, including improved C-reactive protein levels and patient scores on the pain visual analog scale (VAS). Other inhibitors showed limited efficacy or efficacy only in the early stages of the disease. These results show that, despite increasingly effective treatments for patients with RA, further research is required to develop new targeted therapies [15].

In eukaryotic cells, one-third of proteins are synthesized via the secretory pathway, which begins with the translocation of unfolded proteins from the cytosol to a membrane-bound organelle termed the endoplasmic reticulum. In the ER, secretory proteins are synthesized, modified and folded, allowing the appropriate conformation to be achieved [5,7]. Protein folding is much more error-prone compared to DNA replication, transcription or translation [7]. The maintenance of ER homeostasis is essential for proper control of this process, and its disruption is referred to as ER stress. Causes of ER stress can be hypoxia, viral infection, Ca^2+^ deficiency or changes in glycosylation. The accumulation of unfolded or misfolded proteins then occurs, the further development of which is blocked by a signaling pathway termed the unfolded protein response pathway, or UPR [5]. UPR-mediated lysosomal degradation, ERAD—endoplasmic reticulum-associated degradation—and autophagy enhance the efficiency of the removal of misfolded proteins. In order to stop the accumulation of unfolded proteins, the transcription of secretory proteins is decreased. In addition, increased synthesis of foldases and chaperones results in improved protein folding efficiency by the ER [5,8].

In our previous study, it was reported that ER stress that contributes to the activation of UPR may be associated with RA. We found overexpression of eIF2α and PERK in RA patients [16]. Other studies have shown that increased levels of eIF2α and PERK expression are observed in the macrophages and synovial tissues of patients diagnosed with RA. Additional evidence of UPR activity in patients with RA comes from a study by Clavarino G. et al. In this study, increased expression of GADD34, which is involved in the PERK-eIF2a-ATF4 signal transduction pathway, was observed and, in this case, the tested material was PBMCs [17]. 

PERK is a type 1 transmembrane protein that initiates the ER stress response by phosphorylating eukaryotic translation initiation factor 2 subunit-α (eIF2α) at serine 51. The result is a reduction in protein synthesis and, therefore, a reduction in the amount of protein in the ER [5,7]. eIF2α is responsible for initiating translation of specific mRNA fragments containing open reading frames in their non-translating regions [18]. The expression of one of these in response to stress leads to the synthesis of the transcription factor ATF4. It is responsible for inducing the expression of genes involved in amino acid metabolism, redox homeostasis, autophagy and apoptosis. ATF4 participating in a feedback mechanism leads to the dephosphorylation of eIF2α [19]. This results in an increase in protein synthesis through activation of the protein phosphatase 1 (PP1) regulatory subunit GADD34. Under ER stress conditions, a complex of GADD34 with PP1 is formed, resulting in eIF2α dephosphorylation. In addition, there is also a constitutive repressor of eIF2α phosphorylation (CReP), which acts as a PP1 cofactor. Both GADD34 and CReP are involved in the activation of protein synthesis following ER stress inhibition [7].

A cell initiates intracellular apoptotic signaling in response to a stress event [20]. In contrast to necrosis, which is a form of cell death that results from acute cellular injury, apoptosis is a highly regulated and controlled process. The intrinsic pathway is activated by intracellular signals generated when cells are stressed and depends on the release of proteins from the intermembrane space of the mitochondria The extrinsic pathway is activated by extracellular ligands binding to cell-surface death receptors, which leads to the formation of the death-inducing signaling complex (DISC) [21]. During intrinsic apoptosis, cytochrome c is released from the mitochondria through the actions of the BAX proteins [22,23]. Once cytochrome c is released, it binds with Apoptotic protease activating factor–1 (Apaf-1), which then binds to pro-caspase-9 to create a protein complex known as an apoptosome. The apoptosome cleaves the pro-caspase to its active form of caspase-9, which in turn cleaves and activates pro-caspase into the effector caspase-3 [24]. These changes induce cell shrinkage, nuclear DNA fragmentation and mRNA decay. The pro-apoptotic proteins BAX and BAK also activate caspase-3 and induce apoptosome formation during ER stress, thus playing an important role as mediators of ER stress-induced apoptosis [25]. 

In our previous study, we investigated the expression level of mRNA from blood samples of RA patients as well as controls of the unfolded protein response UPR-associated genes using real-time qPCR. The expression levels of *PERK* and its directly acting translation factor *eIF2α*, as well as pro-apoptotic *ATF4* and *BAX* genes, were found to be significantly increased in the blood of RA patients compared with those of the control group [16]. In order to validate our findings, we added new data of molecular markers for ER stress. We determined the expression level of the *eIF2α* factor as a key marker of ER stress and apoptosis factors *PUMA* and *TP53* as executors of cellular stress response. In the present study, the *p*-value for the *eIF2α* was 0.001903, the *p*-value for the *BBC3* (*PUMA*) was 0.007457 × 10^−7^, and the *p*-value for the *TP53* gene was 0.002212. It is suggested that phosphorylated eIF2α could be responsible for the blockade of protein translation in the initiation stage of ER stress. Most importantly, we demonstrated an increased level of the *eIF2α*, which confirmed the dependence of this process on the UPR. Thus, it can be concluded that the components of the UPR-dependent signaling pathway were activated in RA patients.

The TP53 upregulated modulator of apoptosis (PUMA), also referred to as Bcl-2-binding component 3 (BBC3), is a pro-apoptotic protein encoded by the *BBC3* gene [26]. Its expression is controlled by the tumor suppressor TP53. Upon activation, PUMA interacts with anti-apoptotic proteins, leading to the release of Bax, which subsequently transmits apoptotic signals to the mitochondria. In our prior research, Bax expression was identified as an indicator of ER stress in RA patients [16]. Mitochondrial dysfunction then triggers the caspase cascade, ultimately resulting in cell death [27]. Recent findings regarding PUMA indicate that suppressing macrophage-driven inflammation may help restore synovial homeostasis, potentially enhancing the effectiveness of RA therapy [28].

Additional research has demonstrated that PUMA interacts with anti-apoptotic Bcl-2 family proteins, including Bcl-xL and Bcl-2, preventing their association with pro-apoptotic molecules Bak and Bax [29]. In our study, we also examined *Bcl-2* expression in the context of ER stress [16]. When this inhibition is lifted, Bax undergoes translocation, triggering mitochondrial dysfunction, which in turn leads to the release of mitochondrial apoptogenic proteins such as cytochrome c and an apoptosis-inducing factor, AIF. This cascade ultimately results in the activation of specific caspases, including caspase-3, and promotes cell death [26]. Due to PUMA’s strong affinity for Bcl-2 family proteins, another proposed mechanism suggests that PUMA may directly activate Bax and/or Bak, leading to Bax multimerization, mitochondrial translocation and subsequent apoptosis induction [30,31].

Caspase-3 is a caspase protein encoded by the *CASP3* gene, playing a crucial role in apoptosis. It is activated through both extrinsic (death ligand) and intrinsic (mitochondrial) pathways [7]. In the extrinsic pathway, activation initiates a characteristic caspase cascade, where caspase-3 serves as a key regulator of apoptosis [32]. Within the intrinsic pathway, mitochondrial cytochrome c interacts with caspase-9, apoptosis-activating factor 1—Apaf-1—and ATP to facilitate the processing of pro-caspase-3 [33]. Recent studies have explored the potential of mitigating rheumatoid arthritis by inhibiting tumor necrosis factor-induced caspase-3 activity [34].

In this study, we examined ER stress by evaluating the expression of Eukaryotic Initiation Factor 2, a key molecular marker of translation inhibition in the UPR. eIF2α is a crucial initiation factor necessary for most forms of eukaryotic translation initiation. Substantial evidence suggests that the activation of UPR signaling pathways and subsequent eIF2α phosphorylation play a fundamental role in UPR regulation. This process leads to a reduction in overall protein synthesis while selectively promoting the translation of specific mRNAs, including activating transcription factor ATF4 [35]. Generally, ATF4 regulates a wide range of genes, which play a crucial role in cell adaptation to stress conditions, but during long-term ER-stress, ATF4 may also stimulate protein CHOP, which is responsible for the initiation of the apoptotic cascade [36]. eIF2 alfa interacts with the kinase PERK, resulting in the activation of pro-apoptotic Bcl-2 family members by the PUMA downstream pathway and caspase-3 activation [37]. A key role in this process is played by the mitochondria-dependent intracellular pathway of apoptosis activation [38].

The majority of PUMA-induced apoptosis occurs through the activation of the tumor suppressor protein TP53. TP53 is stimulated by survival-related signals, such as glucose deprivation, leading to increased expression of PUMA, a response commonly associated with ER stress [39]. Elevated PUMA levels trigger apoptosis by disrupting mitochondrial function. Additional agents that trigger TP53-dependent apoptosis include proteasome inhibitors and transcription or translation inhibitors, both of which play a crucial role in UPR-dependent activation [40,41]. However, PUMA-induced apoptosis can also occur independently of TP53 activation, initiated by various factors such as oncogenic stress [42], cytokine and kinase dysfunction, immune modulation, and ER stress-related redox imbalance [43]. As a result, ER stress has been explored as a potential therapeutic strategy for RA patients [44].

While DNA damage and nucleus fragmentation may be associated with apoptosis induction, in the present study we showed that PBMC cells from RA patients were more sensitive to DNA damage induced by hydrogen peroxide. There were no differences in the percentage of tail DNA for the level of endogenous DNA damage (*p*-value = 0.11) measured by the comet assay method. However, in patients in whom apoptosis was induced by hydrogen peroxide, the percentage of DNA contained in the tail was 37.78% higher in RA patients than in the control group (*p*-value = 0.0003). Then, examining exogenous damage caused by hydrogen peroxide, we found statistically significant differences between the population of RA patients and the control group (*p*-value = 0.0028). Based on the results, the caspase 3 level was calculated to be 40.17% higher in RA patients than in controls.

Since it was found that PBMC cells from RA patients were more sensitive to apoptotic induction, our results were confirmed by flow cytometry. The statistically significant differences were demonstrated in the percentage of cells in the stage of early apoptosis in RA patients than controls caused by hydrogen peroxide. Moreover, the percentage of healthy cells was higher in the control group than in the RA patients, while there were no differences in the cell viability at the starting point (no hydrogen peroxide). We estimated that, the percentage of healthy cells was higher in controls than the RA population (*p*-value = 0.0003) after hydrogen peroxide incubation. The most important data confirmed elevated sensitivity to apoptosis induction in RA patients with a 40.23% higher percentage of cells in early apoptosis than in the control group (*p*-value = 0.0105).

In connection to our study, it was reported that the PERK- eIF2α UPR pathway might influence the expression of ATF4, which is responsible for activating the translation of the GADD34 and pro-apoptotic factor CCAAT/enhancer-binding protein homologous protein (CHOP). GADD34 controls protein phosphatase 1 (PP1) to dephosphorylate eIF2α, thereby reactivating mRNA translation [45]. PP1 is specific for phosphorylated eIF2α under ER stress conditions in the presence of the cofactor, called the constitutive repressor of eIF2α phosphorylation (CReP). CHOP stimulates ER stress-induced apoptosis due to its ability to modify death receptor 5 (DR5), GADD34 and Bcl-2 family proteins, resulting in an increase in protein synthesis and therefore an increase in the number of misfolded proteins [19].

IRE1α is also classified as a type 1 transmembrane protein containing an endoribonuclease domain and a serine/threonine protein kinase domain [5]. This protein is present in all tissues except mucosal tissue, where a second isoform of IRE1 kinase, termed IRE1β, is found [8]. The accumulation of unfolded proteins causes the ER chaperone BiP to be released from the luminal domain of IRE1α, resulting in its activation. The IRE1α UPR via activation of the signaling pathway TRAF2-JNK or regulated IRE1-dependent decay (RIDD) is involved in caspase 8-dependent, caspase 2-dependent or BAK/BAX-dependent apoptosis. RIDD is also involved in the regulation of thioredoxin interacting protein (TXNIP), resulting in the stimulation of inflammasome, caspase 1 and IL-1β and leading to inflammation and the activation of apoptosis. It should also be noted that the interaction of Ca^2+^ released from the ER via inositol 1,4,5-trisphosphate receptor (IP3R) with BAX inhibitor 1 (BI-1) and GRINA proteins present in the ER results in BAK/BAX-dependent activation of the apoptosome and the release of reactive oxygen species from the mitochondrion [46].

Finally, our data suggested that disruption in the level of apoptosis-related factors may affect the survival of PBMC cells in RA patients, leading to chronic inflammation. Our research team examined the expression of subsequent Bcl-2 family apoptotic genes related to the UPR pathway, and their elevated expression were observed. Studies by independent teams showed that interleukin-17 increases apoptotic genes expression in synoviocytes from RA patients, indicating a potential role in the future pro-inflammatory response [47,48]. Overall, the role of apoptosis induction in RA requires further investigation, but it is suggested that this phenomenon might be associated with ER-dependent response to cellular stress [49].

## 4. Materials and Methods

### 4.1. Study Group

Overall, the research encompassed a group of 61 individuals. The study group comprised 31 patients with diagnosed rheumatoid arthritis, selected from patients at Vadimed Medical Center in Krakow, Poland. The patients were recruited randomly between 2021 and 2024. Concurrently, the control group included 30 volunteers selected from healthy subjects admitted to the Medical Center for other reasons (patients admitted for routine dental check-ups or prophylactic cervical cytology) not associated with chronic inflammatory, cancer or neurodegenerative disorders. All stages (blood sampling for genetics, acute phase reactants and clinical examination) took place on the same day. The control group was matched to the study group regarding sex and age, including 20 women and 10 men. The average age of women in the control group was 58.4, and the average age of men was 62. Blood samples were collected from both groups of patients and controls. The study received approval from the Institutional Bioethics Committee (protocol no. 2/KBL/OIL/2024). All participants provided written informed consent to participate in the study. Prior to commencing the experiments, all participants underwent comprehensive medical examinations. 

Study participants were patients from the outpatient clinic with rheumatoid arthritis newly diagnosed (period of time from the first outpatient visit to the diagnosis of RA was up to a month) by one rheumatologist. The diagnoses also fulfilled the ACR/EULAR criteria. The inclusion criteria were as follows: diagnosis of rheumatoid arthritis based on established diagnostic criteria, age > 45, stable health status, ability to provide informed consent and willingness to follow research procedures, including taking medications regularly. The exclusion criteria were as follows: age < 45, presence of other autoimmune diseases, recent use of immunosuppressives medications, inability to provide informed consent and unwillingness to follow research procedures. The Disease Activity Score (DAS28) was also analyzed, which is commonly used in clinical practice to assess disease activity and joint damage. All patients were naive to treatment and were treated according to current ACR/EULAR recommendations. 

The patient group included 21 women and 10 men; the average age among women was 64 years, while among men it was 69 years. Positive family history of inflammatory joint diseases was present in eight women and 10 men. All the patients were treated with csDMARDs. Short-term oral glucocorticoid therapy was administered to 11 women and nine men during the initiation or modification of csDMARDs. The mean erythrocyte sedimentation rate (ESR) before treatment initiation in the female group was 44.1 mm/h [5–10 mm/h], whereas in the male group it was 42 mm/h. The mean C-reactive protein (CRP) level before treatment initiation in the female group was 33 mg/L [0–5 mg/L], while among men it was 34 mg/L. The mean DAS28 score 3 months after treatment initiation was 2 among women and 2 among men, indicating the achievement of remission. After treatment initiation, a significant decrease in inflammatory parameters was also observed. In the female group, the mean ESR was 15 mm/h, while in the male group it was 11 mm/h. The mean CRP level among women was 6 mg/L, while among men it was 2 mg/L. Based on DAS28 criteria 11 women and four men achieved complete remission, 6 women and six men achieved disease regression and 2 women were referred to biological treatment due to poor response for csDMARDs, Table 1. 

### 4.2. Isolation of PBMCs

Blood collected from patients was suspended in EDTA tubes. Then, the isolation of PBMCs (peripheral blood mononuclear cells) began. For this purpose, the blood was suspended in a DPBS solution (DPBS, no calcium, no magnesium, Gibco™ REF 14190-144) in a 1:1 ratio, then gently applied to Histopaque solution (Histopaque^®^-1077, Sigma-Aldrich, Darmstadt, Germany) in a 1:2 ratio. The prepared samples were centrifuged and then the cells suspended in the interphase were collected. After washing the obtained cells twice with DPBS solution, cell concentration and their viability were counted. For this purpose, trypan blue (Trypan Blue #1450021, BioRad, Hercules, CA, USA) and a cell counter (TC20TM Automated Cell Counter, BioRad, Hercules, CA, USA) were used.

### 4.3. Gene Expression

Blood collected on sodium citrate was refrigerated, then RNA was isolated using a RiboPure™ RNA Purification Kit (AM1928). Using a SYNERGY Microplate reader from BioTek (Santa Clara, CA, USA) the concentration of RNA samples was measured. The next step was to perform the reverse transcription reaction using a High Capacity cDNA Reverse Transcription Kit at an RNA concentration of 20 ng/µL. In the next step, a real-time PCR reaction was performed using TaqMan Universal Master Mix II, TaqMan Gene Expression Assays and EURx nuclease-free water. The qPRC reaction was carried out in a CFX Real-Time System from BioRad (Hercules, CA, USA). The obtained results were analyzed by calculating the Ct values for each gene and the ΔCt values for each gene, respectively, using the *GAPDH* gene as a reference gene. The 2^−ΔCt^ values for the test genes were then calculated.

### 4.4. Induction of Apoptosis

In order to induce apoptosis, PBMC suspensions with a concentration of 1 × 10^6^ were created. The next step was to add hydrogen peroxide solution (Hydrogen peroxide solution, 216763 Sigma-Alrdich, Darmstadt, Germany) to obtain a cell suspension with a H_2_O_2_ concentration of 50 μM. Cells treated in this way were incubated on ice for 20 min. After incubation, cells were washed with DPBS solution to stop further induction of apoptosis by hydrogen peroxide.

### 4.5. Measurement of Caspase 3 Activity

To measure the activity of caspase 3 in cell suspensions, a Caspase-3 Assay Kit (Colorimetric) (AB39401, Abcam, Cambridge, UK) was used, and the manufacturer’s instructions were followed. Absorbance measurement was performed using Multiskan TM SkyHigh with Touch Screen (REF A51119600 ThermoFisher, Waltham, MA, USA).

### 4.6. Measurement by Flow Cytometry

To measure the level of apoptosis in cells, an FITC Annexin V Apoptosis Detection Kit 1 (556547, BD Pharmingen TM, Franklin Lakes, NJ, USA) was used. After analyzing the procedures recommended by the manufacturer, they were changed. After centrifuging the initial cell suspension, the supernatant was poured off and the cells were suspended in 100 μL of 1X binding solution (component no. 51-66121E). Then, 5 μL of FITC Annexin V (component no. 51-65874X) was added, followed by incubation for 15 min at room temperature protected from light. After incubation, 1 mL of 1X binding solution (component no. 51-66121E) was added and centrifuged at 600× *g* for 5 min. The supernatant was poured off, and the cells were suspended in 200 mL of 1X binding solution (component no. 51-66121E), to which 5 μL of Propidium Iodide (PI) (component no. 51-66211E) was added. The solutions prepared in this way were incubated for 15 min at room temperature, protected from light. After incubation, cytometric measurements were made using ZE5 Cell Analyzer (BioRad, Hercules, CA, USA).

### 4.7. Comet Assay

The comet assay was performed under alkaline conditions essentially according to the procedure of Singh et al. [30] with some modification [31]. A freshly prepared cell suspension in 0.75% low-melting-point agarose (UltraPure™ Low Melting Point Agarose, ThermoFisher, Waltham, MA, USA) dissolved in DPBS (DPBS, no calcium, no magnesium, Gibco™ REF 14190-144) was placed onto microscope slides pre-coated with 0.5% normal-melting agarose (UltraPure™ Agarose, ThermoFisher). The cells were then lysed for 1 h at 4 °C in a buffer consisting of 2.5 M NaCl, 100 mM EDTA, 1% Triton X-100, 10 mM Tris, pH 10. After the lysis, the slides were placed in an electrophoresis unit, and DNA was allowed to unwind for 20 min in an electrophoretic solution containing 300 mM NaOH, 1 mM EDTA, pH > 13. Electrophoresis was conducted at room temperature for 20 min at an electric field strength 0.73 V/cm (30 mA). The slides were then stained with 2 μg/mL DAPI and covered with cover slips. Subsequently the slides were examined at 200× magnification in an Eclipse fluorescence microscope (Nikon, Tokyo, Japan) attached to a COHU 4910 video camera (Cohu Inc., San Diego, CA, USA) connected to a personal computer-based image-analysis system, Lucia-Comet (Laboratory Imaging, Prague, Czech Republic).

### 4.8. Statistical Analysis 

Statistical analyses were performed using Statistica 13.1 software (StatSoft, Tulsa, OK, USA). Comet assay results were calculated for each patient by counting 100 randomly selected comet images and calculating the median value of the percentage of DNA contained in the tail. Data on the levels of the caspase 3 and percentage of cells in PBMC populations were presented as means ± SEM (standard error of the mean) of the conducted experiments. The distribution of variables was evaluated using the Shapiro–Wilk test, and statistical analysis of differences between the groups of data was carried out using the Mann–Whitney U test (for non-normal distribution) for both measurement of caspase 3 levels and the percentage of examined cells in PBMC. Values of *p* < 0.05 were regarded as statistically significant (* *p* < 0.05, ** *p* < 0.01 and *** *p* < 0.001).

## 5. Conclusions

RA is one of the most common autoimmune disorders. Due to the heterogeneity of symptoms, patient diagnosis is significantly delayed, which compromises treatment outcomes. This is the reason why research to identify potential diagnostic markers for RA patients is so important [50]. This study showed that the UPR pathway may influence apoptosis and the development of RA. We assume that UPR molecular factors could be potential diagnostic markers or therapeutic targets in ER-related cellular stress in RA patients. This can lead not only to a shorter diagnostic process but also to the discovery of potential targets for personalized therapies via UPR-dependent molecular inhibitors, i.e., PERK-selected inhibitors for further investigations in RA patients. 

## Figures and Tables

**Table 1:** Characteristics of rheumatoid arthritis patients.

Category	Mean Value	SD
Number of patients (n)	31	
Number of patients treated with methotrexate (MTX) (n)	27	
Positive family history (n)	14	
Number of patients bridging with GCS (n)	15	
Number of patients that achieved complete remission (n)	15	
Number of patients with disease regression (n)	14	
Mean ESR before treatment initiation (mm/h)	43.3	17.08
Mean ESR after treatment initiation (mm/h)	13.1	8.64
Mean CRP before treatment initiation (mm/h)	33.15	26.68
Mean CRP after treatment initiation (mm/h)	4.38	5.81
Mean DAS-28 before treatment	4.35	1.18
Mean DAS-28 after treatment	2.39	0.54

Data are presented as means ± SD.

## Data Availability

The data that support the findings of this study are available from the corresponding author upon reasonable request.
